# THE NATIONAL COLLABORATORY TO ADDRESS ELDER MISTREATMENT: COORDINATING NETWORKS OF CARE

**DOI:** 10.1093/geroni/igz038.288

**Published:** 2019-11-08

**Authors:** Kristin Lees-Haggerty, Tony Rosen, Terry Fulmer

**Affiliations:** 1 Education Development Center, Boston, Massachusetts, United States; 2 Weill Cornell Medical College / NewYork-Presbyterian Hospital, New York, New York, United States; 3 The John A. Hartford Foundation, New York, New York, United States

## Abstract

Elder mistreatment is a devastating and pervasive issue affecting an estimated one in ten older adults in the US. To effectively address elder mistreatment, it is essential for researchers, health care providers and communities to coordinate efforts and leverage each other’s strengths. The National Collaboratory to Address Elder Mistreatment (NCAEM), funded by The John A. Hartford Foundation and Gordon and Betty Moore Foundation, is a group of elder mistreatment experts from across the country taking a collective impact approach to alleviating the burden of elder mistreatment on survivors, families, communities, and systems. NCAEM’s goals are to provide hospital emergency departments with training and tools to address elder mistreatment and to develop networks of experts, health care, and community-based providers in a coordinated response to this complex problem. This symposium will include four presentations describing the NCAEM’s collective impact approach, intersection with related work, and applicability to other initiatives. We will present our strategy for creating a team, moving beyond typical collaborative efforts through the development of a central infrastructure and streamlined processes to support experts from across the country as we work together toward common goals and shared measurement for addressing elder mistreatment. Following an overview of our approach, presentations will include specific examples of how the NCAEM worked with clinical partners to refine an elder mistreatment screening and response tool, case examples from NCAEM clinical test sites describing efforts to bridge hospital and community-based services, and learnings from NCAEM’s strategic alignment with complimentary initiatives (the Geri-ED Accreditation and Collaborative).

